# Health-related quality of life in children with developmental coordination disorder: a systematic review

**DOI:** 10.1186/s12955-023-02146-6

**Published:** 2023-06-29

**Authors:** Meyene Duque Weber, Tatiane Targino Gomes Draghi, Liz Araújo Rohr, Jorge Lopes Cavalcante Neto, Eloisa Tudella

**Affiliations:** 1 Department of Physiotherapy, Federal University of Sao Carlos, Sao Carlos, SP Brazil; 2 Department of Human Sciences, Bahia State University, Jacobina, BA Brazil

**Keywords:** Developmental coordination disorder, Health-related quality of life, Children, Systematic review

## Abstract

The aims of this study were (1) to synthesize evidence of the general health-related quality of life in children with DCD compared to their typically developing peers, and (2) to verify which domains of HRQOL are more compromised in children with DCD. A systematic search was carried out to identify cross-sectional studies that evaluated self-perception and/or the parent's perception of the HRQOL in children with and without DCD as an outcome. The methodological quality of the studies was assessed, and the effect size calculated. Initial searches in the databases identified 1092 articles. Of these, six were included. Most of the articles (5/6) included noted that children with DCD show a significantly lower HRQOL than their typically developing peers. Regarding the most compromised HRQOL domains, the results are heterogeneous. Most studies (3/6) had moderate methodological quality, and two studies were classified as high methodological quality. Effect sizes ranged from low to high.

## Introduction

According to the World Health Organization’s (WHO) classification of functionality and health, child development is interdependent, with bidirectional interactions among body structures and functions, activities, participation, and contextual factors (environmental and personal) [1]. When these interactions are positive, they create ideal conditions for movement experiences, providing school-age children whit a broad repertoire of motor skills [2, 3].

Developmental coordination disorder (DCD), which affects 6% of children worldwide, can impact functionality by limiting the acquisition and improvement of motor skills [4]. Individuals with DCD have impaired motor task performance, and may have deficits in balance, coordination, and manual dexterity [4–6]. Diagnosis is based on four criteria, according to Diagnostic and Statistical Manual of Mental Disorders – Fifth Edition Text Revision (DSM-V-TR®): (A) motor performance below expected for chronological age; (B) impaired motor skills interfere with home and daily school activities of the child; (C) motor skill deficits in early developmental period; and (D) deficits not inherent to neurological conditions or visual or intellectual impairment [4].

Children with DCD need a higher level of concentration and more anticipatory and reactive motor adjustments to maintain motor control [7, 8]. However, these adjustments may be insufficient to efficiently refine motor skills, leading to frustration due to the difficulty to perform activities at the same level as their peers. Consequently, fatigue from motor overload, associated with feelings of failure, perpetuates a recurring cycle of motor task avoidance (both individual and in groups), low frustration tolerance, lack of motivation, and decreased self-esteem [9]. In this regard, health-related quality of life (HRQOL) may also be compromised in children with DCD once is characterized by physical, emotional, and social well-being [9–11]. However, there is no universally accepted definition – as the definition of health and quality of life driven by the WHO, neither the differences among health, quality of life, and HRQOL [12, 13]. Nevertheless, HRQOL is widely recognizes as a multidimensional construct and a crucial, useful indicator of individuals' health, and their physical, emotional, and social well-being [12, 14, 15]. HRQOL is typically evaluated using various self-perception indicators of health and functionality, resulting in a comprehensive assessment of how individuals’ well-being or health issues influence their quality of life [16]. The most common domains addressed by standard HRQOL assessment tools for children include physical and emotional/psychological well-being, social support (from family and friends), school environment, and autonomy [17–19].

This concept of HRQOL emphasizes not only the health status but also various domains of a child's environmental factors and life circumstances [11]. Although child health goals and personal expectations are important, family has a fundamental role in the health care process, maintaining consistent attention to promote well-being [20]. The World Health Organization Quality of Life (WHOQOL-100) is an instrument that measures quality of life beyond the absence of disease and encompasses domains concerning physical, psychological, independence, social relationships, environmental, and personal beliefs [11]. Although more questionnaires for specific populations and objectives were developed after WHOQOL-100, there is a lack in the literature of instruments to assess HRQOL in children with DCD and knowledge regarding essential domains (e.g. participation in school activities involving gross and fine motor skills) in these individuals. For this reason, HRQOL is assessed in children with DCD using instruments validated for general populations, with specific domains addressing biopsychosocial and functional aspects.

Zwicker et al. [10] conducted a systematic review that included studies assessing the physical, psychological, and social domains of quality of life in children with DCD. Although the majority of these studies did not consider quality of life as an outcome, they found that children and adolescents with DCD had lower quality of life compared to typically developing (TD) children [10]. Despite the relevance, most included studies did not use quality of life assessment instruments [10]. Consequently, the understanding of these aspects remains limited, as the evidence regarding DCD and HRQOL is constrained by the currently small number of studies available. It is worth noting that the knowledge regarding the relationship between HRQOL domains and DCD should be strengthened by further research, and an update of this systematic review over the years is recommended. Furthermore, the review [10] was conducted in 2012, when diagnostic criteria for DCD were based on DSM-IV®. New evidence considering HRQOL as an outcome among school-aged children with DCD also justifies a new and updated literature synthesis.

Therefore, this systematic review aimed (1) to synthesize evidence of the general health-related quality of life in children with DCD compared to their typically developing peers, and (2) to verify which domains of HRQOL are more compromised in children with DCD. Clarifying impairments in children with DCD beyond motor impairments may provide tools for health and educational professionals to help these children deal with their disorder.

## Material and methods

This systematic review followed the Preferred Reporting Items for Systematic Reviews and Meta-Analyses (PRISMA) [21] recommendations and was based on Cochrane Handbook [22]. The review was registered on PROSPERO platform under identification number CRD42020208819.

### Database and keywords

A literature search was conducted between September and December 2020 in PubMed, PEDro, Web of Science, Scopus, Scielo, and LILACS databases. In January 2022, a new search was conducted in the same databases to recover possible recent papers. Descriptors "Developmental Coordination Disorder", "Motor Skills”, "Child”, and "Quality of Life" were combined using the Boolean operator “AND”.

### Eligibility criteria

Articles were selected according to the following eligibility criteria: (1) original articles published with children (four to 12 years old) with DCD and related terms (probable DCD [p-DCD] or risk for DCD); (2) original cross-sectional, case–control, or cohort observational studies published in Portuguese, English, or Spanish between January 1st, 2010, and January 1st, 2022; and (3) articles assessing self-report or proxy-report by parents regarding HRQOL in children with DCD and TD using instruments developed to assess HRQOL. PICO strategy was used to define eligibility criteria (Table 1).

**Table 1:** Descriptions of Population, Intervention, Comparison, and Outcome (PICO) strategy

PICO Elements	Description
Population	Male and female children, 4 to 12 years of age, with Developmental Coordination Disorder (DCD), probable DCD (pDCD) or at risk for DCD
Intervention/Exposure	Motor deficits related to DCD
Comparison	Male and female children, 4 to 12 years of age, typically developing (TD)
Outcome	Quality of life

### Data extraction and analysis

Two independent reviewers (first and second authors) performed the initial searches, and disagreements were solved by consensus. When necessary, a third reviewer (third author) was consulted. The process began with a screening of titles for duplicates, followed by abstract and full-text reading, considering eligibility criteria.

The following data were analyzed for the narrative synthesis: study design, population characteristics, age, DCD diagnosis, motor test, cutoff points, HRQOL assessment instruments, and main findings. Statistical differences (*p*-value) in overall HRQOL scores between children with DCD and TD were considered.

Mean and standard deviation of HRQOL scores were used to calculate Cohen’s d effect size [23] between children with DCD and TD. Results were interpreted as small (d = 0.20—0.49), moderate (d = 0.50—0.79), or large (d ≥ 0.80) effect [23].

A meta-analysis was not feasible due to the heterogeneity of instruments used, the absence of mean and standard deviation values for HRQOL, and the moderate methodological quality observed in most of the included studies.

### Methodological quality assessment

Methodological quality was assessed using the Newcastle–Ottawa scale (NOS) adapted for cross-sectional [24] and cohort studies [25]. NOS has a star system for scoring studies according to specific criteria for each study design. Cohort studies can receive up to nine stars, based on selection (up to four stars), comparability (up to two stars), and results (up to three stars) [25]. Cross-sectional studies can receive up to ten stars, based on selection (up to five stars), comparability (up to three stars), and outcome (up to two stars) [24]. Methodological quality was considered high (seven stars or higher), moderate (between five and six stars), or low (less than five stars) [24, 25].

## Results

**Fig. 1:**
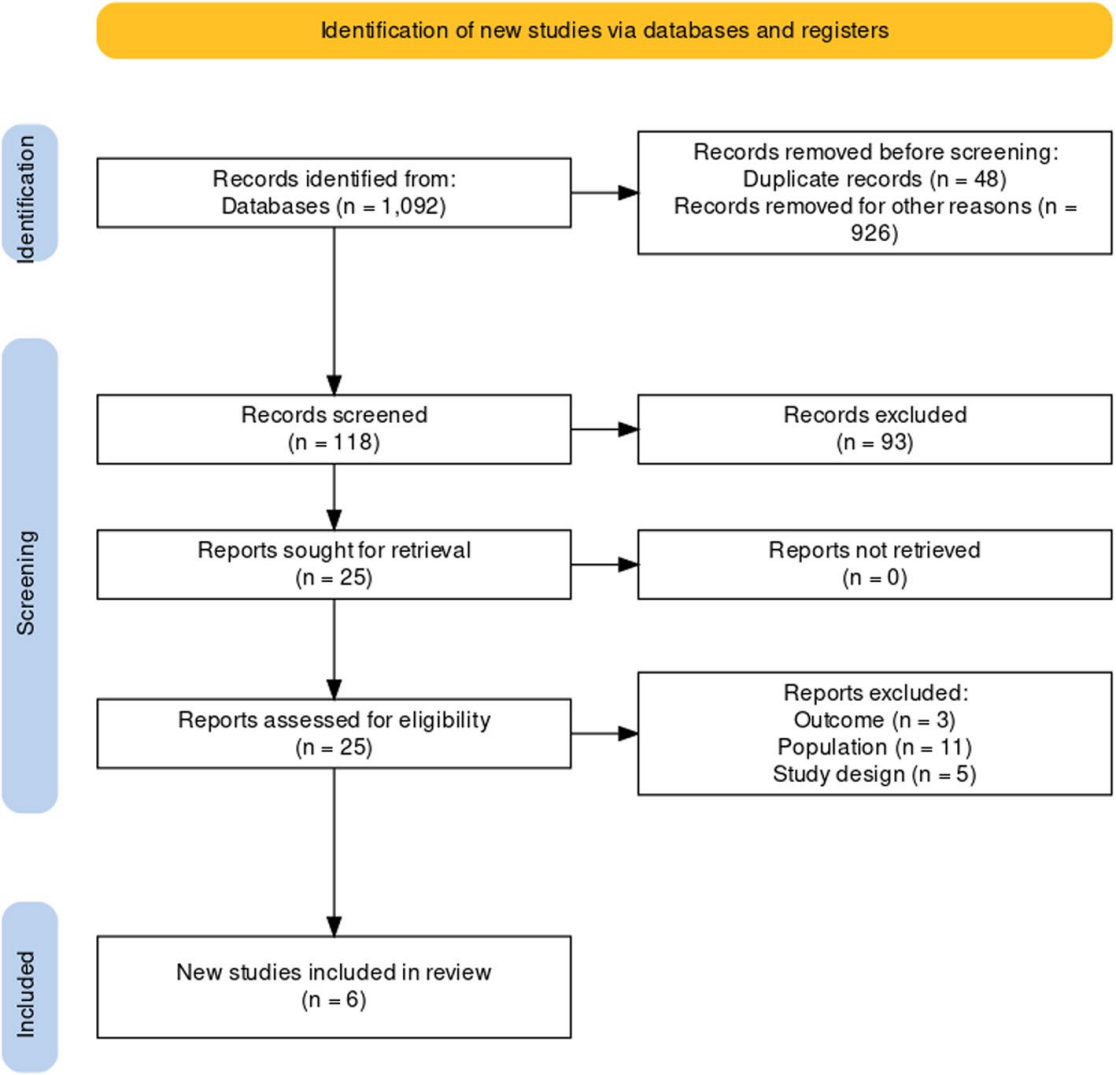
Flow diagram showing the steps in the systematic review *From: Haddaway, N. R., Page, M. J., Pritchard, C. C., & McGuinness, L. A. (2022). PRISMA2020: An R package and Shiny app for producing PRISMA 2020-compliant flow diagrams, with interactivity for optimised digital transparency and Open Synthesis Campbell Systematic Reviews, 18, e1230. *https://doi.org/10.1002/cl2.1230

One thousand ninety-two articles were identified in the initial search. Six articles were included after titles, abstracts, and full-text reading (Fig. 1).

Five cross-sectional studies [26–30] and one cohort [31] were included. Low HRQOL scores were observed in children with DCD compared with TD (the lower the score, the worst the HRQOL). The total sample size was 576 individuals; of those, 225 were children with DCD or p-DCD, 281 were TD, and 70 were guardians. Two articles [27, 28] considered normative samples of instruments as a parameter for TD group; therefore, 49,113 children of normative samples were excluded in this systematic review. Children with DCD and TD aged between four and 12 years. One study [28] used all DSM-V® criteria for DCD diagnosis; two studies [26, 29] did not describe DCD diagnosis; and other three [27, 30, 31] used only “motor assessment” criteria of DSM-V®, performed by a qualified health professional. The Movement Assessment Battery for Children-2 test was used for motor assessment in three studies [28, 30, 31]; this information was not described in the other studies [26, 27, 29]. KIDSCREEN was the most used HRQOL assessment instrument [27–29]. Children with DCD presented lower HRQOL (*p* < 0.05) than TD (Table 2). The effect size was calculated in three out of six studies and ranged between small and large (Cohen’s d 0.46 – 6.37).

**Table 2:** Summary of results from the studies included

Study	Design	Sample	Characteristics of participants			Age group (Range)	Inclusion criteria for DCD	Motor perfor-mance assess-ment	Quality of life assessment	Results regarding the total score of quality of life	*p* value	Effect size (Cohen’s *d*)
			**DCD Termi-nology**	**DCD group (N)**	**TD group (N)**							
**Kennedy-Behr et al., 2015 **[26]	Cross-sectional	63	pDCD	32 and their parents	31 and their parents	4 – 6 years	ND	ND	KINDL	No significant differences were observed between children with and without DCD	*p* = 0.08	*0.46*
**Caçola & Killian, 2018 [**27]	Cross-sectional	96	DCD	96	Normative sample	6 – 12 years	DCD diagnosis given by a qualified health professio-nal	ND	PedsQL	Children with DCD had significantly lower quality of life than TD children	*p* < 0.001	*2.02*
**Caçola & Killian, 2018 **[27]	Cross-sectional	96	DCD	96	Normative sample	6 – 12 years	DCD diagnosis given by a qualified health professional	ND	KIDSCREEN	Children with DCD had significantly lower quality of life than TD children	*p* < 0.001	*-*
**Karras et al., 2018 **[28]	Cross-sectional	100	DCD	50 children with DCD and their parents (50)	Normative sample	8 – 12 years	DSM-V: (a) MABC-2; (b) DCDQ; (c) e (d) by interview	MABC-2	KIDSCREEN-52	Children with DCD had significantly lower quality of life than TD children	*p* < 0.005	-
**Ganapathy Sankar & Monisha, 2020 **[29]	Cross-sectional	42	DCD	10 children with DCD and their parents (10)	12 children with TD and their parents (10)	5 – 10 years	ND	ND	KIDSCREEN-27	Children with DCD had significantly lower quality of life than TD children	*p* = < 0.001	-
**Redondo-Tébar et al., 2021 **[30]	Cross-sectional	115	DCD	19	96	4 – 7 years	MABC-2	MABC-2	KINDL	Children with DCD had significantly lower quality of life than TD children	*p* = *0.02*	*6.37*
**Uusitalo et al., 2020 **[31]	Cohort	160	DCD	18	142	11	MABC-2 and The Touwen Neurological Examination	MABC-2	17-Dimensional Illustrated Questionnaire (17D)	Children with DCD had significantly lower quality of life than TD children	*p* = 0.03	*-*

*DCD* Developmental Coordination, *TD* Typical development, *ND* Not described, *pDCD* Probable DCD, *DSM-V* Diagnostic and statistical manual of disorders, Fifht Edition. *MABC-2* Movement Assessment Battery for Children-2, *DCDQ* Developmental Coordination Disorder Questionnaire, *KINDL* Revised Children Quality of Life Questionnaire. 17D: 17-Dimensional Illustrated Questionnaire

Children with DCD presented lower scores on HRQOL domains than TD group. Most compromised domains were pre-school [26], peers and social support [27], social [27], moods and emotions [28], physical well-being [29], hearing [30], and friends [31] (Table 3).

**Table 3:** DCD/pDCD and TD groups punctuations in quality of life domains of the presented instruments

Study	Domains	DCD/pDCD Group M (SD)	TD Group M (SD)
**Kennedy-Behr et al., 2015 **[26]	Preschool	75.40 (10.77)*	86.20 (10.09)*
**Caçola & Killian, 2018 **[27]	Peers & Social Support	38.63 (15.04)*	50 (ND)*
	Social	41.09 (0.85)*	79.51 (20.73)*
**Karras et al., 2018 **[28]	Moods & Emotions	46.8 (10.15)*	52.2 (9.97)*
**Ganapathy Sankar & Monisha, 2020 **[29]	Physical Well-Being	42.09 (ND)*	ND*
**Redondo-Tébar et al. (2021) **[30]	Friends	79.3 (3.0)*	87.6 (1.3)*
**Uusitalo et al., 2020 **[31]	Hearing	92 (ND)*	98 (ND)*

*M* Mean, *SD* Standard deviation, *DCD* Developmental Coordination Disorder, *TD* Typical development, *ND* Not described, *pDCD* Probable DCD

^*^p < 0.05

Three studies had moderate methodological quality [26–28] and scored 50% of NOS items. Two studies were classified as high methodological quality, scoring 77% of items [31] and 80% of items [30], while another study with low methodological quality [29] scored only 10% on NOS (Table 4). Sample size, non-respondents, determination of exposure (risk factor), and comparability between children with DCD and TD were the least scored items.

**Table 4:** Study quality assessment using Newcastle–Ottawa Scale for cross-sectional and cohort studies

**Study**	**Design**					**NOS**					
		**Selection 1**	**Selection 2**	**Selection 3**	**Selection 4**	**Comparability 1a**	**Comparability 1b**	**Outcome 1**	**Outcome 2**	**Outcome 3**	**NOS total score (%)**
**Kennedy-Behr, et al. (2015) **[26]	Cross-sectional	*	NA	NA	NA	*	*	*	*	-	5/10 (50%)
**Caçola & Killian (2018) **[27]	Cross-sectional	*	*	*	NA	NA	NA	*	*	-	5/10 (50%)
**Karras et al. (2018) **[28]	Cross-sectional	*	NA	NA	**	NA	NA	*	*	-	5/10 (50%)
**Ganapathy Sankar & Monisha (2020) **[29]	Cross-sectional	NA	NA	NA	NA	NA	NA	*	NA	-	1/10 (10%)
**Redondo-Tébar et al. (2021) **[30]	Cross-sectional	*	NA	*	**	*	*	*	*	-	8/10 (80%)
**Uusitalo et al. (2020) **[30]	Cohort	*	*	*	*	*	*	NA	NA	*	7/9 (77%)

*NOS* Newcastle–Ottawa Scale

^*^one star. *NA* Not available

## Discussion

The present systematic review synthesized existing evidence of the general HRQOL in children with DCD compared to their typically developing peers, and (2) verified which domains of HRQOL are more compromised in children with DCD. Five out of six studies [27–31] reported lower HRQOL scores in children with DCD compared to TD children.

### HRQOL in children with DCD versus TD children

One study [27] showed children with DCD had lower HRQOL scores than children with chronic health conditions and TD children, as perceived by guardians. In line with this outcome, the study by Redondo-Tébar et al. [30] also revealed lower HRQOL scores in children with DCD compared to TD children, according to parents’ perception [30]. Other three studies [28, 29, 31], identified that children with DCD rated their own HRQOL as lower than that of their TD peers.

One study [26] did not observe differences in HRQOL between children with DCD and TD children, except for one domain. This result explains the perception of guardians who may overestimate their children's well-being. Still, these younger children are experiencing socialization and the real perception of the challenges of the disorder, which may not be noticeable in the caregiver's view. In addition, the criterion for identifying pDCD was not described, which prevents understanding whether such a level of motor difficulty could impact their quality of life.

Furthermore, age (four to six years and 11 months) [26] may have contributed to the findings since self-perception of motor skills performance and social interactions occur at later ages [30, 32, 33].

### HRQOL by guardians’ perception versus children self-report

It is essential to remind that HRQOL, as defined by World Health Organization, is based on an individual’s perception and influenced by several contextual factors, not solely related to health conditions. Therefore, a self-report may offer more accurate insight than a proxy-report by parents, especially during childhood, when most part of their day passes in school and their relationships are built in that environment. According to Lee et al [34], the correlation between HRQOL perceived by children and their parents is a topic that needs to be better understood. Gothwal et al [35] point out that it is unlikely that parents are aware of all their children’s difficulties, since most of the time, these difficulties tend to happen at school and with friends – times when parents are not around. However, there is no consensus in the literature that parents overestimate the HRQOL of their children, and there are studies that do not show this difference between the perception of parents and children [35].

Three studies [28–30] evaluated both guardians’ and children perception of HRQOL. One of these studies [30] identified that self-report HRQOL in children with DCD was lower than TD, but not statistically significant [30], suggesting that parents may underestimate children. The authors [30] suggest that the small sample size (*n* = 115) and the age of the children (under six years old) may explain the results of self-report HRQOL. Other two studies [28, 29] also indicate that the guardians’ perception probable underestimate HRQOL of children with DCD.

One study [31] assessed only self-reported HRQOL in children, while another study [27] assessed only guardians’ perception. Both studies reported lower HRQOL scores for children with DCD compared to TD children.

Therefore, one study [26] that found no difference in HRQOL between children with p-DCD and TD children relied solely on guardians' perceptions. According to guardians, children with p-DCD perform daily life situations similarly to their TD peers [26]. This could suggest that children with p-DCD may not yet have developed the self-awareness to perceive social isolation, reduced self-esteem, and decreased social participation [26], thus, their guardians may not have noticed these behaviors. Although the HRQOL scores were not different between guardians of children with p-DCD and guardians of TD children [26], the results are relevant and reinforce the need for assessing self-perceived HRQOL in children across different age groups.

### Assessment of HRQOL domains among children with DCD and TD children

The most impaired HRQOL domains were pre-school [26], peers and social support [27], social [27], moods and emotions [28], physical well-being [29], hearing [31], and friends [30].

These differences in scores on quality-of-life domains may stem from the heterogeneity of the results, the construction of each domain, and, primarily, the potential protective factors present in children’s daily life contexts, enduring into adult life [36]. With these findings in mind, it is crucial that the care provided to these children is individualized and specific, ensuring that both the assessment and the intervention are directly tailored to each individual's unique needs [37]. Furthermore, PedsQL and KIDSCREEN, being assessments with low to moderate correlations among domains, could potentially explain the observed heterogeneity in the results. Zwicker et al. [10] review also demonstrated variability in the most impaired domains. Given these considerations, it seems reasonable to suggest that there are unique characteristics within HRQOL domains for children with DCD.

Since these factors were not assessed in the studies included in this review, we recommend further investigation taking into consideration factors such as family support, friendships, level of involvement in physical education classes, and other contextual factors that may help children with DCD cope with their constraints.

Findings of each study were heterogeneous for most compromised domains, even using the same assessment instrument. The most used instrument was KIDSCREEN (versions with 27 [27, 29] and 52 items [28]). KINDL [26, 30] was used twice. PedsQL [27] and 17D [31] instruments were used only once.

Domains with the lowest scores in studies that used KIDSCREEN were peers and social support [27], moods and emotions [28], and physical well-being [29]. Social [27], preschool and friends [26, 30], and hearing [31] domains had the lowest scores when assessed using PedsQL, KINDL, and 17D, respectively. Results regarding HRQOL domains for children with DCD were inconclusive, probably due to heterogeneous assessment instruments, diagnostic criteria for DCD, sensorimotor and social experiences of children, selection criteria (i.e., age group, respondents, co-occurrences of other neurodevelopmental disorders in children with DCD), and cultural differences related to the educational system of each country.

### Methodological quality of studies

The methodological quality of the included studies was heterogeneous. Three [26–28] studies presented moderate quality and their findings should be interpreted cautiously, as the authors did not perform selection and comparison criteria, which raises the risk of bias. Three [26, 28, 29] studies did not describe sample selection, justified sample size, or detailed the respondent characteristics. Instruments used for DCD assessment were also not adequately described, raising doubts about the definition of the study population [26, 27, 29]. Although two [27, 28] studies did not control confounding factors when interpreting results (e.g., co-occurrence of dyspraxia and attention deficit hyperactivity disorder or dyslexia), most of the studies [26–28] did include specific outcome criteria (e.g., assessment and analysis of results), reducing interpretation bias.

### Effect size, clinical implications, and study limitations

The effect size was calculated for three [26, 27, 30] studies and was found to be small (d = 0.46) in one [26], and large (d = 2.02 – d = 6.37) in the other two [27, 30]. These findings indicate that HRQOL in children with DCD may be impaired, highlighting the importance of multi-professional assessment and early interventions that consider the biopsychosocial and functional aspects of these children.

Further studies are necessary to better understand HRQOL in children with DCD. Although the effect sizes ranged from small to large, the ages and the HRQOL assessment instruments used in two of the studies [26, 27] were heterogeneous. Nevertheless, Kennedy-Behr et al. [26] and Redondo-Tébar et al. [30] were similar in terms of age groups and HRQOL assessment instruments used. Therefore, clinical interpretation of the impact of DCD on HRQOL in children must be undertaken with caution. Age appears to be a determining factor for HRQOL, as large [27, 30] and small [26] effect sizes were observed in older and younger children, respectively. Guardians perceive increased motor impaired behaviors regarding DCD characteristics, such as enhanced clumsiness, in older children, suggesting that signs of DCD become more apparent with age [38], which can justify why the effect size of the HRQOL results appears to be larger in older children [27, 30] in the studies included in this review. Despite different assessment instruments [26, 27, 30], HRQOL was also evaluated by guardians. Whenever possible, HRQOL should be both evaluated by self-report and proxy-report by parents to increase accuracy. Therefore, a combination of assessments (i.e., self-perception of children and guardians) is recommended to understand HRQOL from various perspectives.

Two studies [26, 29] did not describe the diagnostic criteria for DCD, which does not allow us to know how these children were diagnosed and whether the terminology used follows international recommendation [39]. Moreover, only one study [28] followed the four DSM-5 diagnostic criteria. These gaps identified in the studies regarding diagnosis are limitations that can bias the interpretation that the results are related to children with DCD and their typically developing peers. Although the current evidence from this systematic review does not assure the diagnostic criteria for DCD were followed, low motor proficiency impacted children’s quality of life and should be a concern to be explored in further investigations. Additionally, it is based on what is available in the literature taking into account the difficulty to establish a formal diagnosis where the condition is still unfamiliar.

Children identified with DCD in the included studies show lower overall HRQOL than their typically developing peers. Results are inconclusive for HRQOL domains due to methodological heterogeneity between studies. The findings of this systematic review need to be interpreted with caution due to the moderate methodological quality observed in most studies. Primary studies strictly following methodological recommendations are needed to avoid bias and elucidate HRQOL domains in children with DCD.

## Data Availability

The datasets used during the current study are available from the corresponding author on reasonable request.

## Abbreviations

DCD: Developmental Coordination Disorder
HRQOL: Health-related Quality of Life
TD: Typically developing
NOS: Newcasttle-Ottawa Scale
pDCD: Probable Developmental Coordination Disorder
DSM-V: Diagnostic and statistical manual of mental disorders – Fifht Edition
MABC-2: Movement Assessment Battery for Children-2
DCDQ: Developmental Coordination Disorder Questionnaire
KINDL: Revised Children Quality of Life Questionnaire
17D: 17-Dimensional Illustrated Questionnaire
DSM-V-TR: Diagnostic and Statistical Manual of Mental Disorders – Fifth Edition Text Revision
